# What do medical faculty students think about telehealth?

**DOI:** 10.12669/pjms.40.8.9055

**Published:** 2024-09

**Authors:** Memet Taşkın Egici, Seda Ozmen Sever, Guven Bektemur, Güzin Zeren Ozturk, Hasan Bagcı, Mulazim Hussain Bukhari

**Affiliations:** 1 Memet Taşkın Egici Associate Professor, Head of Family Medicine Department, Istanbul Haydarpaşa Numune Education and Research Hospital, University of Health Sciences, Istanbul, Turkey; 2 Seda Ozmen Sever Family Medicine Specialist, Department of Family Medicine, Istanbul Şişli Hamidiye Etfal Education and Research Hospital, University of Health Sciences, Istanbul, Turkey; 3 Güven Bektemür Associate Professor, Deputy Dean of Hamidiye Faculty of Medicine, Department of Public Health, University of Health Sciences, Istanbul, Turkey; 4 Güzin Zeren Oztürk Associate Professor, Department of Family Medicine, Istanbul Şişli Hamidiye Etfal Education and Research Hospital, University of Health Sciences, Istanbul, Turkey; 5 Hasan Bağcı Associate Professor, Freelance Researcher, Health Management, Ankara, Turkey; 6 Mulazim Hussain Bukhari Professor, Department of Pathology, Principal Azad Jammu Kashmir Medical College Muzafarrabad, Muzafarrabad, Pakistan

**Keywords:** Distance learning, Mobile health, Medical student, Medical education, Telemedicine, Telehealth

## Abstract

**Objective:**

We aimed to evaluate medical school students’ knowledge and approaches regarding telehealth.

**Method:**

In this descriptive study, an electronic survey was conducted among students attending the Faculties of Medicine at Health Sciences University. The first part of the Form included questions evaluating individual characteristics and telehealth approaches, and the second part included opinions and suggestions regarding telehealth usage topics.

**Results:**

Of 698 participants, 435 (64.9%) students were in the preclinical period. One hundred nine (15.6%) believed they had sufficient knowledge about telehealth while 399 (57.2%) believed that telehealth should be included in medical education. When asked about their opinions on using telehealth in their professional careers, 298 (42.7%) stated that they considered using them. Those who perceived themselves as having sufficient knowledge about telehealth were more inclined to consider using it more in their professional careers (p=0.000). Participants who believed that healthcare services could be provided through telehealth were more likely to think that disease monitoring would be better, patient follow-up quality would improve, unnecessary hospital admissions would decrease (p<0.05).

**Conclusions:**

The majority of medical faculty students lack sufficient knowledge about telemedicine and believe that education on this topic should be included in the medical curriculum. It is suggested that incorporating pre-clinical courses on telemedicine and providing internship opportunities in practical settings would effectively address this gap.

## INTRODUCTION

The ongoing advancements are widespread in their application of technology in various aspects of life today, facilitating ease of access and operation in transportation and beyond. The World Health Organization (WHO) defines telemedicine as the provision of healthcare services using information and communications technologies (ICTs) in various areas such as diagnosis, treatment, prevention of diseases and accidents, research, assessment, health education, and health improvement, particularly when distance is a critical factor.^1^,^2^

Telehealth has a wide range of applications, not only limited to accessing remote patient data and monitoring but also extending to activities like prescribing medication and performing surgeries. It is widely acknowledged that this broad spectrum of applications can enhance the quality and efficiency of healthcare services.^3^ The inception of telehealth dates back to the early 20th century. While historical records indicate that the initial applications were pioneered by a Dutch cardiologist, it gained widespread use, particularly in the United States and Canada, primarily in the field of radiology, in the subsequent years.^4^

On the basis of the Health Transformation Program launched in 2002 in Turkey, the electronic recording system “E-pulse”, through which physicians can access patient medical records via mobile application after the person’s approval, was implemented by the Ministry of Health (MoH) in 2015.^5^ Before the pandemic, in 2019, the “Telehealth and Teleradiology Directive” was issued, focusing on the transmission, archiving, and remote reporting of radiological images.^6^,^7^ However, with the onset of the pandemic, telehealth began to take a more prominent role in practice; consultations and examinations were conducted via phone calls and online platforms and in early 2022, MoH published more detailed regulation about remote healthcare services.^8^,^9^

Telemedicine applications facilitate access to healthcare services, shorten appointment times, support healthcare professionals’ access to people and data, and help reduce workload. As a result, it is expected to increase support for patients and their families, improve the quality of chronic disease care, and reduce healthcare costs.^10^-^13^ The increasing prevalence of telemedicine applications requires users, especially physicians, to become familiar with these applications. In this study, we aimed to evaluate medical school students’ knowledge and approaches regarding telehealth.

## METHOD

This is a single-center, descriptive study which included students Faculties of Medicine at Health Sciences University. There were a total of 3516 students in attendance, and the sample size was determined to be 354 with a confidence level of 95%. Medical school education in Turkey is six years; the first three years are considered as the preclinical period, the second three years are considered as the clinical period.

After obtaining consent from the participants, a 15-question data Form prepared by reviewing the literature was administered online. The first section of the data Form included questions assessing individual characteristics and their approaches toward telehealth. The second section addressed their opinions on the areas of telehealth usage, and propositions related to telehealth applications were presented with triple Likert-type responses.

The parameters used in the study were categorized and classified individually. Numeric data were presented with mean and standard deviation, while categorical data were presented with median and percentiles. T-tests were used for comparing numeric data, and chi-square tests were used for comparing categorical data. A significance level of p<0.05 was considered, and the statistical analysis was conducted using the SPSS 25 software package.

### Ethical Approval:

Study approval was received from the Health Sciences University Hamidiye Scientific Research Ethics Committee (Meeting date: 24/02/2023, Decision number: 4/13).

## RESULTS

Our study had a total of 698 participants. Among the participants, males accounted for the majority with 79.9% (n=558). The majority of the participating students were in the preclinical period (n=435, 64.9%). One hundred nine (15.6%) believed they had sufficient knowledge about telehealth while 301 (43.1%) believed that healthcare services could be implemented through telehealth. Additionally, 399 (57.2%) believed that telehealth applications should be included in medical education.

When asked about their opinions on using telehealth applications in their professional careers, 298 (42.7%) stated that they considered using them, while 269 (38.5%) mentioned that they were unsure about it. As for the belief that telehealth applications would improve access to healthcare and enhance the quality of healthcare services, 65 (9.3%) responded with ‘no,’ and 18 (2.6%) responded with ‘definitely no.’

Participants provided their answers to the question regarding in which areas telehealth can be used; it was believed to be most suitable for prescription and reporting, and preventive medicine Table-I. Among the participants, 60 (8.6%) believed that telehealth was not suitable for any of the mentioned applications, 106 (15.2%) believed that all areas were suitable for use. Out of those who had previously received online healthcare services, 12 (6.7%) stated that the mentioned areas were not suitable for telehealth use.

**Table-I:** Participants’ answers to the question "in which topics is telehealth used?"

	Yes (n/%)	No (n/%)	I have no idea (n/%)
Preventive medicine	504 (72.2%)	74 (10.6%)	120 (17.2%)
Polyclinic services	348 (49.9%)	236 (33.8%)	114 (16.3%)
Inpatient follow-up	321 (46%)	254 (36.4%)	123 (17.6%)
Elderly care / Home healthcare/ Palliative care	441 (63.2%)	147 (21.1%)	110 (15.8%)
Follow-up after treatment	504 (72.2%)	109 (15.6%)	85 (12.2%)
Counseling services (Family therapy, infertility and nutrition)	514 (73.6%)	81 (11.6%)	103 (14.8%)
Consultation, health commission, health reports etc.	377 (54%)	200 (28.7%)	121 (17.3%)
Preoperative evaluation	272 (39%)	311 (44.6%)	115 (16.5%)
Surgical/interventional procedures and postoperative follow-up	263 (37.7%)	312 (44.7%)	123 (17.6%)
Management of complications (with remote surgery)	293 (42%)	271 (38.8%)	134 (19.2%)
Triage before emergency services	345 (49.4%)	233 (33.4%)	120 (17.2%)
Prescription and health report writing	529 (75.8%)	84 (12%)	85 (12.2%)

The distribution of participants’ responses to statements regarding telehealth applications is presented in Table-II. The statement that received the highest agreement was “There will be frequent technical problems in telehealth use”. On the other hand, the statement that received the least agreement was” Telehealth should only be used in extraordinary situations like disasters or pandemics”. It has been stated that there may be problems in terms of data security and privacy for a significant number of participant (55.4%).

**Table-II:** Distribution of participants’ responses regarding their thoughts on telehealth applications.

	I agree (n/%)	I don’t agree (n/%)	I have no idea (n/%)
“Absence of face-to-face clinical evaluation in telehealth services will have a negative impact.”	396 (56.7%)	100 (14.3%)	202 (28.9%)
“Physician’s lack of clinical knowledge and experience will have a negative impact on the use of telehealth.”	384 (55%)	90 (12.9%)	224 (32.1%)
“With telehealth, disease monitoring, patient follow-up quality and patient compliance with treatment will be better.”	365 (52.3%)	100 (14.3%)	233 (33.4%)
“Telehealth reduces unnecessary hospital admissions and laboratory tests.”	406 (58.2%)	92 (13.2%)	200 (28.7%)
“The possibility of malpractice increases in telehealth use.”	331 (47.4%)	99 (14.2%)	268 (38.4%)
“There will be frequent technical problems in telehealth use (internet access, audio-visual quality, etc.)”	460 (65.9%)	56 (8%)	182 (26.1%)
“Telehealth can cause privacy and data security problems.”	387 (55.4%)	110 (15.8%)	201 (28.8%)
“Telehealth should only be used in extraordinary situations like disasters or pandemics”	274 (39.3%)	163 (23.4%)	261 (37.4%)
“E-pulse application is sufficient to support telehealth in Turkey”	276 (39.5%)	142 (20.3%)	280 (40.1%)

When examining the relationship between participants’ perception of having sufficient knowledge about telehealth and their socio-demographic characteristics, it was found that male students and those in the preclinical period believed they had sufficient knowledge about telehealth (respectively; p:0.000, p:0.000). Additionally, those who perceived themselves as having sufficient knowledge about telehealth were more inclined to consider using it more in their professional careers (p:0.000). (Table-III)

**Table-III:** Comparison of the answers about telehealth according to the preclinical/clinical periods.

		Preclinical period (n/%)	Clinical period (n/%)	p
I have used the telehealth service before.	Yes	136 (30%)	44 (18%)	0.001
	No	317 (70%)	201 (72%)	
I have sufficient knowledge for telehealth.	Yes	81 (17.9%)	28 (11.4%)	0.000
	No	178 (39.3%)	135 (55.1%)	
	Undecided	194 (70.3%)	82 (33.5%)	
Telehealth should be included in medical education curriculum.	Yes	268 (59.2%)	131 (53.5%)	0.274
	No	61 (13.5%)	42 (17.1%)	
	I have no idea	124 (27.4%)	72 (29.4%)	
Health services can be provided through telehealth.	Yes	213 (47%)	88 (35.9%)	0.005
	No	119 (26.3%)	91 (37.1%)	
	I have no idea	121 (26.7%)	66 (26.9%)	
Thanks to telehealth, diseases can be diagnosed earlier.	Definitely yes	88 (19.4%)	37 (15.1%)	0.068
	Yes	181 (40%)	83 (33.9%)	
	Undecided	149 (32.9%)	97 (39.6%)	
	No	25 (5.5%)	23 (9.4%)	
	Definitely no	10 (2.2%)	5 (2%)	
Telehealth facilitates access to healthcare services and improves the quality of healthcare.	Definitely yes	108 (23.8%)	42 (17.1%)	0.022
	Yes	180 (39.7%)	85 (34.7%)	
	Undecided	119 (26.3%)	81 (33.1%)	
	No	34 (7.5%)	31 (12.7%)	
	Definitely no	12 (2.6%)	6 (2.4%)	
Telehealth saves time.	Yes	326 (72%)	168 (68.6%)	0.567
	No	50 (11%)	33 (13.5%)	
	I have no idea	77 (17%)	44 (19%)	
Thanks to telehealth, health records are seen by all physicians.	Yes	323 (71.3%)	152 (62%)	0.012
	No	130 (28.7%)	93 (38%)	
I am planning to use telehealth in my professional life.	Yes	210 (46.4%)	88 (35.9%)	0.003
	No	89 (19.6%)	42 (17.1%)	
	I have no idea	154 (34%)	115 (46.9%)	

Participants who had previously used telehealth services believed that disease monitoring would be better with telehealth, patient follow-up quality would improve, and patients’ adherence to treatment would be better. However, they also believed that technical problems would be more frequent in telehealth use (respectively; p:0.004, p:0.047). Additionally, participants who had previously used telehealth services been more inclined to believe that healthcare services could be provided through telehealth with a statistically significant difference (p:0.000).

Participants who believed that healthcare services could be provided through telehealth were more likely to think that disease monitoring would be better, patient follow-up quality and treatment adherence would improve, unnecessary hospital admissions and tests would decrease, and the e-Pulse system was a sufficiently supportive application for telehealth. These beliefs were statistically significantly higher (respectively; p:0.000, p:0.002, p:0.000). On the other hand, those who believed that healthcare services could not be provided through telehealth were more likely to think that the absence of face-to-face clinical evaluation in telehealth use would have a negative impact. They also believed that a lack of clinical knowledge and experience on the part of the physician, an increased likelihood of malpractice, technical problems, and potential issues with privacy and data security could arise in telehealth use. These beliefs were statistically significantly higher (respectively; p:0.000, p:0.000, p:0.022, p:0.001, p:0.013).

In our study, male participants and those in the preclinical period were considering using telehealth in their professional careers (respectively; p:0.000, p:0.003). Regarding the areas where telehealth could be used, preclinical period students believed with a statistically significant difference that it could be used in inpatient follow-up, preoperative evaluation, and surgical/interventional procedures, as well as in postoperative follow-up (respectively; p:0.000, p:0.047, p:0.000).

## DISCUSSION

In our study, when looking at the participants’ responses regarding the areas of telehealth use, they indicated that the most common areas would be preventive medicine, counseling, and prescription services, where patients are not physically examined, and any diagnosis and treatment are not administered. We believe that concerns such as the absence of face-to-face clinical assessment, the negative impact of knowledge and skill deficiency, data and security problems, and the increased possibility of malpractice may be the reasons for this.

The advancement of technology has led to an increase in the use of online applications in the field of healthcare. The opinions of medical school students regarding telehealth are important for its future use and for its inclusion in the medical education curriculum.

In a study assessing the awareness of telehealth among doctors and medical students in Turkey, it was found that awareness of telehealth was at a moderate level.^14^ This indicates the need to add courses related to telehealth to the medical education curriculum. It may be necessary to incorporate telehealth as a separate internship/course within the medical school curriculum and allow students to practice through telehealth, considering the expected increase in the use of this system in the future.

In a study conducted in the United States, which involved an assessment of third-year students’ knowledge of telehealth before and after a specific education, 80% of the students stated that they could use telehealth in the future.^15^ In another study conducted with nursing and medical students, participants believed that e-health applications would contribute to healthcare services, improve health outcomes, and lead to changes in the daily routines of healthcare professionals.^16^ Approximately half of the participants in this study stated that they considered using telehealth applications in their professional careers, and it was found that those who were knowledgeable about telehealth were more inclined to consider its use. Additionally, the majority of students believe that telehealth usage will facilitate access to healthcare services and improve their quality.

On the other hand, telehealth applications increase close monitoring of patients and can reduce unnecessary hospital admissions. In a review conducted in Turkey in 2019 regarding the importance of nursing care in postoperative telehealth management, it was stated that postoperative remote healthcare services can lead to a reduction in surgical site infections, long-term follow-ups for patients with postoperative complications can be provided, applications such as monitoring drains and vital signs can be performed for patients, and as a result, patients can be protected from complications to a greater extent. This can lead to a decrease in hospital admissions and an increase in the bed occupancy rate.^17^

When looking at the negative views about telemedicine in the literature, it is observed that legal and ethical issues are predominantly addressed. Among these, concerns related to ensuring patient privacy are highlighted.^18^-^20^ Additionally, in some studies, it has been noted that telemedicine may not be suitable for situations requiring a physical examination.^21^ In a study conducted by Holtz to assess telemedicine satisfaction, it was found that all participants were generally satisfied with their telemedicine experiences.^22^

When looking at the concerns of students who want to use telehealth in their professional lives, it can be observed that the absence of face-to-face assessment, potential problems arising from lack of knowledge and experience, the possibility of malpractice, technical issues, as well as privacy and data security concerns are prominent. On the other hand, the majority believed that the use of telemedicine can save patients time, would reduce healthcare costs, provide quality medical care for chronic illnesses.^23^

Medical education is focused on training competent physicians through a combination of theoretical and practical instruction. The mentor-mentee relationship holds significant importance in medical education and, despite the opportunities presented by telehealth, the absence of physical examination and the reliance on senses, even down to recognizing the feelings of patients, emphasize the enduring importance of hands-on medical practice in the art of medicine.

## CONCLUSION

The majority of medical faculty students lack sufficient knowledge about telemedicine and believe that education on this topic should be included in the medical curriculum. It is suggested that incorporating pre-clinical courses on telemedicine and providing internship opportunities in practical settings would effectively address this gap. While telemedicine is considered practical and convenient, students are aware of technological and legal issues and acknowledge that it cannot fully replace in-person examinations. Furthermore, it is important to teach students about the development of telemedicine, provide practical training, and raise awareness about its mandatory role in the future of healthcare delivery.

### Authors’ Contributions:

**MTE**, **SO**, **GB**, **GZO**, **HB** and **MHB:** Conceived, designed.

**MTE**, **SO**, **GB**, **GZO** and **HB:** Did data collection and manuscript writing.

**MTE**, **SO**, **GZO** and **HB:** Did statistical analysis.

**MTE**, **SO**, **GB**, **GZO**, **HB** and **MHB:** Did review, editing and final approval of manuscript.
